# Adjuvant Role of CT in the Diagnosis of Post-Infarction Left Ventricular Free-Wall Rupture

**DOI:** 10.4021/cr239w

**Published:** 2012-11-20

**Authors:** Jorge A Brenes, Terry Keifer, Rehan M Karim, Gautam R Shroff

**Affiliations:** a Division of Cardiovascular Diseases, Mayo Clinic, Rochester MN, USA; b Department of Radiology, Hennepin County Medical Center, Minneapolis, MN, USA; c Divsion of Cardiovascular Diseases, Hennepin County Medical Center, Minneapolis, MN, USA

**Keywords:** Myocardial infarction, Shock, Ventricular rupture, Computed tomography

## Abstract

Left ventricular free wall rupture is usually a catastrophic mechanical complication of myocardial infarction. Risk factors include advanced age, female gender and absence of prior infarction. The vast majority of patients succumb rapidly due to cardiac tamponade and electromechanical dissociation. Expedited and accurate diagnosis can improve the chances of survival. Echocardiography has been advocated as the gold standard for diagnosis, but other imaging modalities can provide valuable information in these patients. We present the case of a patient who presented with cardiogenic shock, in which the definitive diagnosis of a left ventricular free wall rupture was accomplished by CT scan with intravenous contrast.

## Introduction

Mechanical complications of myocardial infarction continue to challenge physicians in the modern reperfusion era. Amongst these, left ventricular free-wall rupture (LVFWR) can be responsible for as much as 20-30% of all infarct-related deaths [1]; it carries a particularly grim prognosis, due to the precipitous progression of cardiogenic shock in most patients. Prompt, specific diagnosis can provide an opportunity for surgical intervention. Transthoracic echocardiography is extremely useful in this setting, due to real-time acquisition of images, bedside availability and high sensitivity for detection of pericardial effusions. However, when aortic dissection is a competing diagnosis, CT with intravenous contrast and delayed images can differentiate both. We present the case of a postmenopausal patient who suffered a cardiac arrest in the setting of a probably silent recent myocardial infarction, in which CT yielded the specific diagnosis and location of a LVFWR.

## Case Report

**Figure 1:**
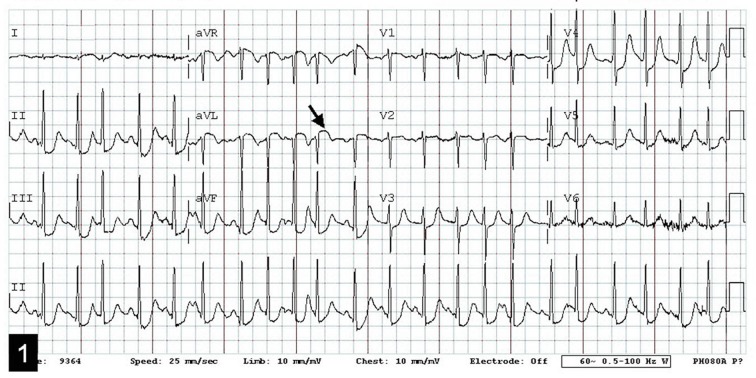
ECG on presentation showing subtle ST segment elevation (arrow) and a deep-Q wave in AVL, along with marked ST segment depression in inferior leads.

**Figure 2:**
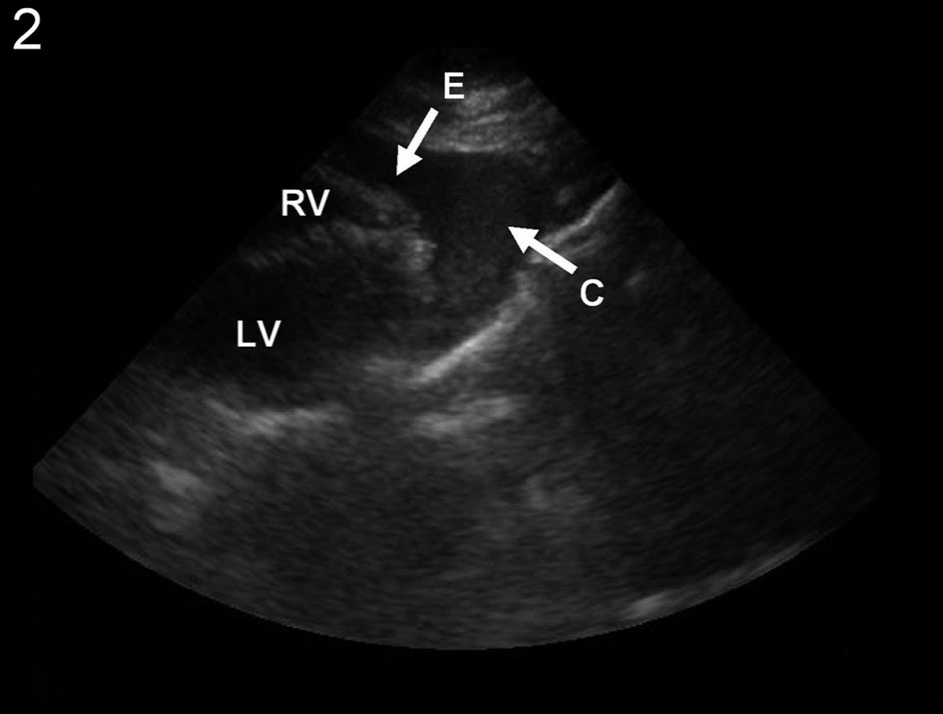
Emergent bedside echocardiogram in the subcostal projection, showing the presence of a moderate pericardial effusion (E), along with clot (C) suggesting hemopericardium. RV: right ventricle; LV: left ventricle.

**Figure 3:**
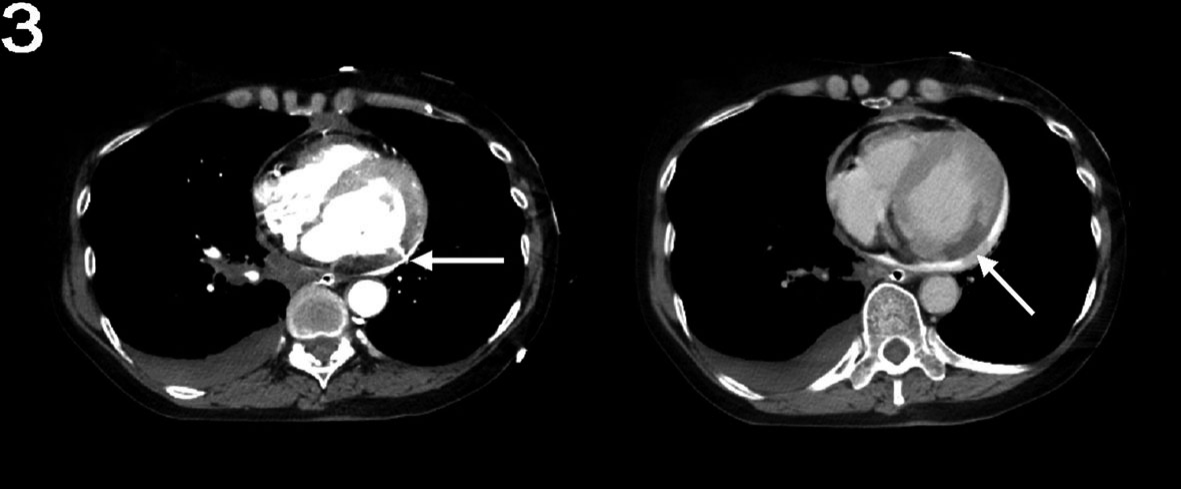
Left: Early images with a CT scan displaying a small rupture (arrow) in the posterolateral wall of the left ventricle with the presence of a hemopericardium, confirming the diagnosis of free wall rupture. Right: Delayed CT images (60 seconds after initial images) showing a small, hypodense area of infarction (arrow) in the inferolateral wall, with greater degree of contrast extravasation relative to the early images, suggesting active bleeding into the pericardial sac.

A 65-year-old female with a history of COPD and tobacco use but no previously documented coronary artery disease was transported to the Emergency Department after a witnessed cardiac arrest. Her husband reported that the patient suddenly clutched her chest in pain and collapsed. She had been complaining of increasing fatigue and malaise for 2 days. The patient was found to have pulseless electrical activity by EMS, but had return of spontaneous circulation after CPR en route. On arrival, she was in shock. Physical examination did not reveal elevated neck veins or rales on auscultation. Initial ECG (Fig. 1) showed a deep Q-wave in lead AVL, with discrete ST-segment elevation, as well as significant ST-segment depression in infero-lateral leads. Chest x-ray indicated widening of the mediastinal silhouette. Limited bedside echocardiogram (Fig. 2) revealed a moderate-sized pericardial effusion with free-floating thrombus, suggestive of hemopericardium. An emergent computed tomography (CT) of the chest was performed to exclude acute aortic dissection, while the operating room was being mobilized. The CT did not reveal any evidence for aortic dissection. However, a site of cardiac rupture through the posterolateral wall of the left ventricle was demonstrated (Fig. 3), and the presence of hemopericardium was corroborated. Additional delayed images were obtained 1 minute later, which revealed a greater degree of contrast extravasation, confirming the presence of active bleeding within the pericardial sac. Moreover, the actual site and small size of the myocardial infarction was also confirmed, being hypodense relative to the remainder of the myocardium. The patient was taken to the operating room and was found to have a large hemopericardium arising from a LVFWR. Despite emergent surgical intervention, the patient did not survive.

## Discussion

More than 350 years after the first description of a LVFWR by William Harvey [2], it is still a diagnosis made more frequently at autopsy. Its estimated clinical incidence according to the SHOCK registry [3] is 2.7% of those patients presenting with an acute myocardial infarction (AMI) and cardiogenic shock; nevertheless, this may be an underestimation, since probably a vast majority of patients die immediately without confirmation of the cause of death. This is suggested by a significantly higher prevalence of this condition (30.7%) in autopsy reports of patients dying from acute myocardial infarction [4]. LVFWR after an AMI can be classified into acute and subacute depending on clinical presentation. The acute type is characterized by abrupt hemodynamic collapse and electromechanical dissociation, which result in unsuccessful advanced cardiac life support measures and death within minutes or hours. The subacute variety, which can represent up to one third of the cases [1], presents in a less dramatic way, with symptoms and signs of a worsening pericardial effusion; in this instance, formation of a hematoma at the epicardial surface can decrease the rate of formation of hemopericardium and provide a time window of opportunity for diagnosis and definite management.

From an imaging standpoint, a new pericardial effusion is the most common finding, and can be quickly assessed by an echocardiogram. The absence of an effusion by echocardiography in an individual with an AMI virtually excludes the diagnosis of myocardial rupture [5], but its presence as an isolated feature in such patients does not definitely confirm the diagnosis, as direct signs of rupture are only occasionally seen with this technique [6]. Even with transesophageal echocardiogram, the sensitivity for detection of LVFWR is about 70% [7]. This poses an additional diagnostic challenge, since the two most frequent causes of lethal hemopericardium are LVFWR and intrapericardial extension of an ascending aortic rupture [8], and both can present with chest pain and hemodynamic collapse. Emergent CT scan with intravenous contrast is able to differentiate them, accurately excluding aortic dissection and confirming the site of myocardial rupture, typically as a slit-like defect through the ventricular wall [9, 10]; furthermore, enhanced-delayed images can be obtained rapidly and allow detection of active extravasation. It also has the potential advantage, as demonstrated in our case, to detect a clinically overlooked AMI, by features such as low density of infarcted myocardium on pre-contrast images and hypo-enhancement or paradoxical hyper-enhancement on post-contrast images [11].

Awareness of the risk factors associated with post-infarction LVFWR is important to maintain a high-degree of suspicion in the appropriate clinical circumstances. These include age over 65, female gender, hypertension without significant left ventricular hypertrophy and late presenters [12]. Diabetes, prior myocardial infarction and peripheral vascular disease are significantly less prevalent in patients with LVFWR and shock, and pulmonary edema is less frequently found on physical examination [3]. It has been hypothesized that these comorbidities could confer a relative “protection” against myocardial rupture due to increased left ventricular mass and fibrosis. The patient presented in our case fit most of these demographic and clinical features, except for a prior diagnosis of hypertension.

The most typical ECG sequence described for patients presenting with the acute-type of LVFWR after an AMI is that of anterior ST segment elevation followed by sudden bradyarrhythmia or electromechanical dissociation [13]; however, in the sub-acute type the ECG is often non-diagnostic. The pattern seen in our case, with a deep Q-wave in AVL, and marked inferior ST segment depression was unusual, but has been described in prior reports [14].

### Conclusions

LVFWR continues to be an ominous complication of acute myocardial infarction, that should be strongly suspected in patients presenting with cardiogenic shock, particularly when other risk factors such as advanced age, female gender, absence of prior diabetes, coronary or peripheral vascular disease are noted on initial assessment. Echocardiography remains a key modality in the expedited diagnostic sequence required in these cases, but emergent CT with contrast can be an adjuvant tool in confirming the diagnosis and excluding other causes of hemopericardium. Further studies are needed to compare the sensitivity and specificity of these imaging modalities in patients with LVFWR.
